# Combined uncertainty estimation for the determination of the dissolved iron amount content in seawater using flow injection with chemiluminescence detection

**DOI:** 10.1002/lom3.10057

**Published:** 2015-09-18

**Authors:** Geerke H. Floor, Robert Clough, Maeve C. Lohan, Simon J. Ussher, Paul J. Worsfold, Christophe R. Quétel

**Affiliations:** ^1^ Institute for Reference Materials and Measurements, Joint Research Centre–European CommissionGeelBelgium; ^2^ Biogeochemistry Research Centre, School of Geography, Earth and Environmental SciencesPlymouth UniversityPlymouthUnited Kingdom; ^3^ Present address: GFZ German Research Centre for Geosciences, Helmholtz Centre PotsdamPotsdamGermany

## Abstract

This work assesses the components contributing to the combined uncertainty budget associated with the measurement of the Fe amount content by flow injection chemiluminescence (FI‐CL) in <0.2 *μ*m filtered and acidified seawater samples. Amounts of loaded standard solutions and samples were determined gravimetrically by differential weighing. Up to 5% variations in the loaded masses were observed during measurements, in contradiction to the usual assumptions made when operating under constant loading time conditions. Hence signal intensities (V) were normalised to the loaded mass and plots of average normalised intensities (in V kg^−1^) vs. values of the Fe amount content (in nmol kg^−1^) added to a “low level” iron seawater matrix were used to produce the calibration graphs. The measurement procedure implemented and the uncertainty estimation process developed were validated from the agreement obtained with consensus values for three SAFe and GEOTRACES reference materials (D2, GS, and GD). Relative expanded uncertainties for peak height and peak area based results were estimated to be around 12% and 10% (coverage factor *k* = 2), respectively. The most important contributory factors were the uncertainty on the sensitivity coefficient (i.e., calibration slope) and the within‐sequence‐stability (i.e., the signal stability over several hours of operation; here 32 h). For GD, using peak height measurements, these factors contributed respectively 69.7% and 21.6% while the short‐term repeatability accounted for only 7.9%. Therefore, an uncertainty estimation based on the intensity repeatability alone, as is often done in FI‐CL studies, is not a realistic estimation of the overall uncertainty of the procedure.

The ocean acts as both a sink and a source for carbon dioxide and plays an important role in regulating the global climate system (Boyd and Elwood 2010). The dynamics of the ocean and its interaction with the atmosphere are strongly linked to the properties of seawater. Elements such as Fe limit marine primary production in half of the world ocean (Moore et al. 2001) and thus may have a profound effect on plankton communities and the global carbon cycle (Martin and Fitzwater 1988; Mills et al. 2004). More reliable determinations of micronutrient elements in marine waters are thus essential to enhance our understanding of their impact on ocean productivity and processes (e.g., ocean acidification). Therefore, robust and fully validated measurement procedures are necessary, accompanied by an estimation of the overall uncertainty budget.

The international standard ISO/IEC 17025 (2005) states that the performance of a measurement procedure should be evaluated based on one or a combination of the following approaches: (1) the use of reference materials, (2) the comparison of results achieved with other methods, (3) inter‐laboratory comparison, (4) systematic assessments of the factors influencing the result and (5) the assessment of the uncertainty of the results. The Fe content of commercially available certified reference materials is at least one order of magnitude higher than most open ocean waters and are thus of limited use for method development. Therefore, test materials from inter‐laboratory comparison exercises are often used instead, e.g., those collected as part of the IRONAGES, SAFe, and GEOTRACES studies. However, Bowie et al. (2006) observed that discrepancies between results obtained in different laboratories during the IRONAGES comparison remained too large (e.g., up to 59% variability when using the same procedure) and differed significantly at the 95% confidence level. Factors thought to explain these results included: (1) variations in the efficiency of the extraction of iron from the matrix during pre‐concentration (resulting in different procedures measuring different fractions of iron), (2) errors in the quantification of the analytical blank, (3) inaccuracies in the system calibration and (4) underestimation of the stated uncertainty (Bowie et al. 2003; Petrov et al. 2007). Hence iron data from these exercises for the same water mass were distinctly inconsistent. Points (1) and (2) have been addressed by the SAFe (Johnson et al. 2007) and GEOTRACES (GEOTRACES 2013) exercises but not points (3) and (4). It is thus useful to revisit these two factors and determine how realistic uncertainties can be estimated for the most commonly applied measurement procedures (particularly shipboard procedures) (*see* also Ussher et al. 2010b). In this respect flow injection with chemiluminescence detection (FI‐CL) was chosen for this study as it is a technique that allows high temporal and spatial resolution measurements at sea without the need for sample storage and transport.

According to the international nomenclature, the measurement uncertainty is a “non‐negative parameter characterizing the dispersion of the quantity values being attributed to a measurand, based on the information used” (JCGM 200 2012). The basic purpose of an uncertainty statement is to propose a range of possible “true” values. There are various ways of estimating uncertainties. For instance, combined uncertainty estimates can be based on data obtained by inter‐laboratory or intra‐laboratory studies (*see* e.g., Analytical Methods Committee 1995; Nordic Committee on Food Analysis 1997). The uncertainty estimation proposed in the Guide for Uncertainty in Measurements (GUM) is based on combining the contributions of all known sources of uncertainty (JCGM 100 2008). In this approach, the measurement procedure is described by a mathematical model and the values and associated standard uncertainties of the different components (the input quantities) in the model must be established. The model and input data are then used to calculate the measurement result including its associated combined uncertainty.

The aim of this work was to study the application of the “GUM approach” to the FI‐CL measurement procedure. The specific objectives were to: (1) propose a set of mathematical equations (a model) describing this measurement process and allowing the estimation of a measurement uncertainty, (2) discuss the best way to assess the uncertainties of the different components in the model, (3) apply this uncertainty model to present the measurement results with their estimated combined uncertainties obtained for seawater reference materials from the SAFe and GEOTRACES campaigns (Lohan et al. 2006; Johnson et al. 2007) and, from the above, (4) propose a simplified equation to estimate the measurement uncertainty.

## Materials and procedures

### Reagents, materials and samples

Concentrated hydrochloric acid (HCl), ammonia (NH_3_, 20–22%) and glacial acetic acid (CH_3_CO_2_H), all SpA (Super Pure Acid) grade, were purchased from Romil (Cambridge, UK). Hydrogen peroxide, Merck Suprapur grade was obtained from VWR (Lutterworth, UK). Luminol (5‐amino‐2,3‐dihydro‐1,4‐phthalazinedione), sodium carbonate and triethylenetetramine (TETA) were purchased from Sigma Aldrich (Gillingham, Dorset, UK). All high purity water (HPW), 18.2 MΩ·cm, was drawn from an ElgaStat Maxima system (Marlow, UK). All weighing was performed using an analytical balance (OH1602/C, Ohaus, Thetford, UK). The accuracy of the balance was checked daily before use using F1 Class certified weights (KERN, Albstadt, Germany). All facilities were managed under ISO 9001:2008 certification.

To ensure low blank Fe amount content all sample and reagent handling was undertaken in an ISO 14644‐1 Class 5 laminar flow hood (Bassaire, Southampton, UK) situated within an ISO 14644‐1 Class 5 clean room. Reagent and sample containers were made of low density polyethylene (LDPE; Nalgene, Fisher Scientific, UK) and were cleaned using established cleaning protocols for trace metals. Containers were immersed in ∼1.1 M trace metal grade HCl (Fisher Scientific) for at least 7 d. Subsequently, the containers were rinsed in copious amounts of HPW, filled with 0.01 M HCl and stored in double re‐sealable plastic bags until use.

The main characteristics of the seawater samples used for this project are described in Table 1. Briefly, all samples were filtered at sea and then acidified either at sea or at Plymouth University (PU). Seawater samples, referred to as SWA, SWB, and SWC, containing  ≤ 0.5 nmol kg^−1^ Fe were selected to prepare three different sets of calibration standards, by addition of controlled amounts of iron from a CPI International (Amsterdam, Netherlands) ICP‐MS standard containing 0.17 mol kg^−1^ Fe. Experiments in this work were carried out with 0.5 L reference samples from large volumes of homogenised, bulk seawater samples (SAFe D2 and GEOTRACES GS and GD consensus mean reference materials). More details regarding the sampling, pre‐treatment and bottling procedures for these materials can be found elsewhere (Johnson et al. 2007; GEOTRACES 2013).

**Table 1:** Description of the samples used.

Sample name	SWA	SWB	SWC	SAFe campaign	GEOTRACES campaigns		
				D2‐578	GS‐132	GD‐158	
Collection location	05˚20.5′ S, 06˚11.9′ W to 06˚44.8′ S, 05˚04.8′ W	27° 47.2′ S, 07° 12.9′ W	40° S 48.46° W	30° N, 140° W	31°40′ N 64°10′ W	31°40′ N 64°10′ W	
Depth	Surface	500m	Surface	1000m	Surface	2000m	
Filtration	Sartorius Sartobran‐P cartridge. Cellulose acetate 0.45 *μ*m pre‐filter then 0.2 μm filter	Whatman GD/X PTFE 0.2 µm filter	Pall Acropak Supor capsule. PES 0.8 pre‐filter then 0.2 *μ*m filter	Polycarbonate track etched 0.45 *μ*m pre‐filter, then 0.2‐*μ*m filter. Homogenized in 1000L fluorinated LDPE tanks	Pall Acropak Supor capsule. PES0.8 pre‐filter then 0.2 *μ*m filter		
Acidification	Bulk sample acidified at sea with 700mL of ∼ 10 M Q‐HCl. Homogenized in 1000L fluorinated LDPE tanks	Acidified at Plymouth University (PU) with 1mL of Romil UpA grade HCl per L seawater	Acidified at PU with 2mL of Romil UpA grade HCl per L seawater	Acidified at sea with 2mL of conc HCl per L seawater	Homogenized in 500L fluorinated LDPE tanks. Acidified at sea with 2mL of conc HCl		
Final pH	2.0	2.0	1.7	1.8	1.8		1.8
Salinity	34.1	30.6	34.2		36.7		34.9
Consensus dissolved Fe ± 2s.d. (nmol kg^−1^)	0.53 ± 0.20	N/A	N/A	0.933 ± 0.046	0.546 ± 0.092		1.0 ± 0.2
Reference	Bowie etal. (2006)	–	Wyatt etal. (2014)	Lohan etal. (2006)	Johnson etal. (2007)		

### The FI‐CL based measurement procedure

**Figure 1:**
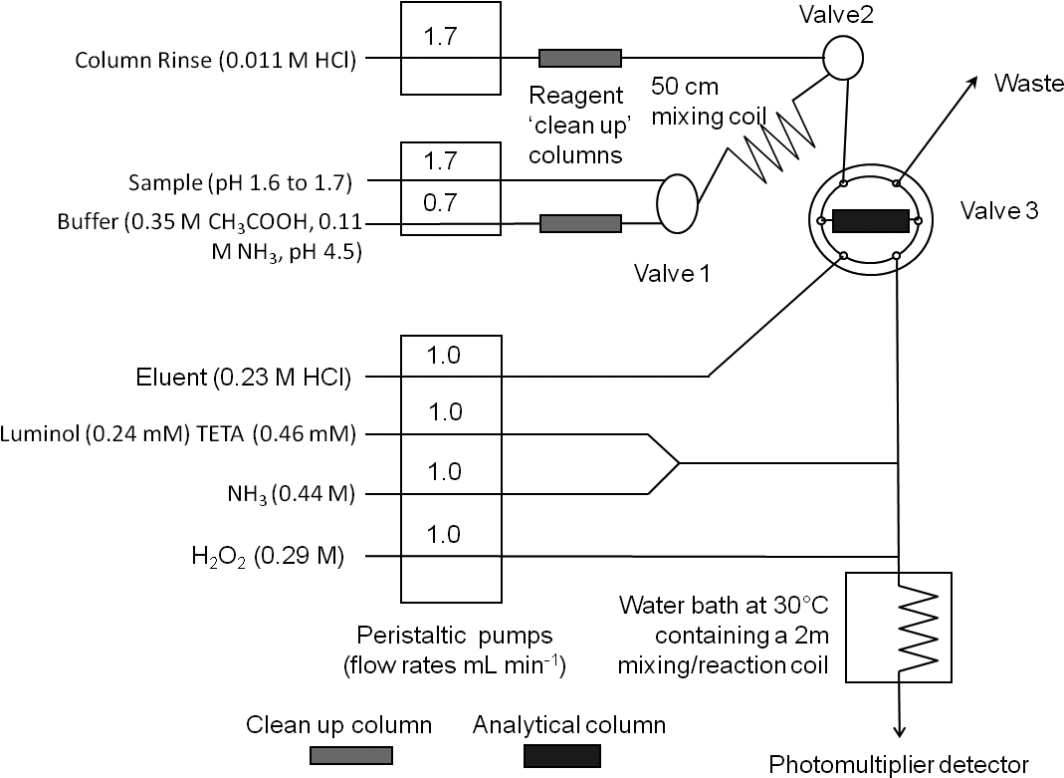
The FI‐CL system used for the determination of dissolved Fe levels in seawater.

Figure 1 describes the FI‐CL manifold used for these experiments. It consists of three peristaltic pumps (Minipuls 3, Gilson, Luton, UK), one PTFE manually operated three port valve (Valve 1; Omnifit), one three port solenoid valve (Valve 2), one two‐way six port electronically actuated valve (Valve 3; VICI, Valco Instruments, Schenkon, Switzerland), a thermostatic water bath (Gran, Cambridge, UK) and a photomultiplier tube (PMT; Hamamatsu H 6240‐01, Hamamatsu Photonics, Welwyn Garden City, UK) containing a coiled, transparent PVC flow cell (volume 40 μL). The peristaltic pump tubing used was two stop Accu‐Rated™ PVC (Elkay, Basingstoke, UK) and all other manifold tubing was 0.8 mm i.d. PTFE. The pumps were turned on and run for 2 h before any measurements were made. If the pump tubing was changed it was conditioned by running the pump slowly overnight. The system used two poly(methyl methacrylate) columns (1 cm long, 1.5 mm i.d., volume 70 μL), loaded with Toyopearl AF Chelate 650 resin (Tosoh Bioscience, Stuttgart, Germany) retained with HDPE frits (BioVion F, 0.75 mm thick, 22–57 μm pore size), to clean up the buffer and column rinse solutions (the clean‐up column on the rinse solution line is not strictly necessary). The analytical column, also loaded with Toyopearl AF Chelate 650 resin, was made of polyethylene with LDPE frits with an internal volume of 200 μL (Global FIA, Fox Island, U.S.A.). Further details of the physico‐chemical properties of the resin can be found in Shelley et al. (2010).

Peristaltic pump, valve control and data acquisition were performed using custom built hardware and software (Ruthern Instruments, Bodmin, UK) run under Labview v 7.1 (National Instruments, Newbury, UK). The measurement procedure, based on the chemistry reported in Obata et al. (1993), was as follows. A working solution of approximately 0.35 μmol kg^−1^ Fe was prepared gravimetrically by serial dilution of the CPI International stock solution. This working solution was then used to gravimetrically prepare calibration standards and achieve added levels ranging from 0.15 nmol kg^−1^ to 0.9 nmol kg^−1^ Fe in 0.15 nmol kg^−1^ increments. All calibration standards were prepared at least 12 h before use to allow for complete equilibration of the added Fe with that present in the calibration seawater. A 20 μL aliquot of a 10 mM H_2_O_2_ solution was added to all calibration standards at least 2 h before use, to ensure that all Fe present was as Fe(III) (Lohan et al. 2006). The following solutions were also prepared at least 12 h before use. A 48 mM stock solution of luminol was obtained by dissolving 0.177 g of luminol and 0.25 g of Na_2_CO_3_ in 20 mL of HPW. This stock was then diluted to give a 0.24 mM working solution. The post column reagents for the chemiluminescence reaction was a mixture of 0.23 M HCl, 0.44 M NH_3_, 0.24 mM luminol/0.46 mM TETA and 0.31 M H_2_O_2_. The acidified reference samples and standards of seawater were buffered on‐line to pH 3.5 with 0.35 M CH_3_CO_2_H and 0.11 M NH_3._ To precondition and wash the column, 0.011 M HCl was used.

To operate the FI‐CL instrument, the LabVIEW software was opened and the baseline signal from the PMT monitored to check for stability. The pump controlling the eluent and post‐column reagents was then activated and the baseline chemiluminescence signal recorded after the signal had stabilised. Each analytical session started with the measurement of a procedural blank (by application of the “closed sample line” method). For this, the sample flow was stopped, by closing one port on valve 1, so that only the rinse solution and ammonium acetate buffer passed over the column. The FI‐CL system was then operated by loading and injecting SWA for at least 30 min to monitor stability. Subsequently, calibration seawater standards and samples were analysed. The FI‐CL manifold was fully automated and one replicate measurement consisted of the following analytical cycle. The column was conditioned for 10 s with 0.011 M HCl. Then the sample and buffer were loaded simultaneously for 60 s. The loading pH was optimised for maximum retention of Fe (Clough et al. 2015). The column was washed with 0.011 M HCl for 20 s. The Fe was then eluted with 0.23 M HCl for 120 s (total time for one analytical measurement = 210 s). Six replicate measurements were made for each sample or standard solution. The mass of loaded sample or standard solution was gravimetrically determined for each replicate by differential weighing. Between each sample the sample flow path was washed with HPW for 30 s followed by uptake of the fresh sample for 180 s to flush the line up to valve 2 (total time for one analytical cycle of measurement and washing for six replicates = 21 min). After each analytical session all fluid paths were flushed with 0.011 M HCl for 10 min and then with HPW for 15 min and HPW was left in the lines.

### Data treatment

Data integration was also performed with the custom build software run in LabVIEW. The baseline, and the start and end points of the peak were set manually for each transient signal. The main calculations in this study were carried out on the basis of peak height data, as this is common practice for FI‐CL measurements in the oceanographic community (and the wider FI community). Peak area measurements were also made and some of the differences observed when using peak areas are discussed below. Further data treatment, including calculations for the estimation of standard uncertainties, was carried out in Excel®. The combined uncertainties were obtained by propagating together individual uncertainty components according to the GUM (JCGM 100 2008). In practice, a dedicated software program was used (Metrodata GmbH 2003). The reported combined uncertainties are expanded uncertainties and reported as *U* = *ku*_c_ where *u*_c_ is the combined standard uncertainty and *k* is a coverage factor equal to 2. If “*the probability distribution characterized by y and u*_c_ *(y) is approximately normal and the effective degrees of freedom of u*_c_ *(y) is of significant size*” (“*greater than 10*”), “*taking k = 2 produces an interval having a level of confidence of approximately 95%*” (JCGM 100 2008).

## Assessment

### Description of the measurand

The GUM states that a measurement begins with an appropriate specification of the measurand, the particular quantity intended to be measured (JCGM 100 2008). Iron exists in different physico‐chemical forms in seawater. Traditionally, filtration is performed to differentiate between the different physical size fractions (Wu et al. 2001; Ussher et al. 2004, 2010a). Additionally, iron occurs in two oxidation states; Fe(II) and Fe(III). Generally, Fe(III) predominates in oxygenated waters, of which most (80–99%) is strongly complexed by organic ligands (Achterberg et al. 2001; Mawji et al. 2008; Gledhill and Buck 2012). In this study, the measurand is the amount content of Fe present in <0.2 *μ*m filtered and acidified samples and is regarded as the dissolved fraction of the Fe present in the seawaters. The aim was to obtain the Fe amount content in specific samples and therefore the uncertainties associated with the sampling process and/or the sample conditioning phase have not been considered.

### Experimental design

Three different types of experiment were performed in different analytical sessions. First, the stability of the analytical procedure was checked with five measurements (six replicates of each) performed over a period of 32 h for SWC and a procedural blank using the closed sample line approach to obtain the within‐sequence stability. This experiment was termed the “stability experiment.” The FI‐CL manifold was run continuously for this period, i.e., the pumps remained on and the same batch of reagents was used throughout. Thirty two hours is the maximum time that the manifold can operate continuously with a single batch of reagents. Second, the effect of small variations in the matrix was investigated by comparing the sensitivity factor using three different seawaters as standards (Table 1). On the first day, SWA was compared with SWB while on the second day SWA was compared with SWC (“matrix experiment”). Third, the FI‐CL based procedure was applied to the determination of iron in samples of three filtered and acidified seawater reference materials using SWA for calibration (“reference material experiment”).

### Calculating the dissolved Fe amount content in the samples and mathematical description of the measurement procedure

Implicit in the GUM “is the assumption that a measurement can be modelled mathematically to the degree imposed by the required accuracy of the measurement” (JCGM 100 2008). A measurand *Y* is determined from various input quantities *X*_i_ through a functional relationship. These input quantities “may themselves be viewed as measurands and may themselves depend on other quantities, including corrections and correction factors” “that can contribute a significant component of uncertainty to the result of the measurement” (JCGM 100 2008). A mathematical description of the FI‐CL measurement procedure is given through Eqs. 1–5 described in Table 2. The main equation in this procedure is the calculation of the dissolved Fe amount content in a sample by dividing the blank corrected sample intensity by the sensitivity of the system (Eq. 1 in Table 2). The way the equations controlling these three input parameters were established is discussed below.

**Table 2:** Mathematical equations for quantification of the Fe amount content using gravimetric loading and FI‐CL based procedure.


1. Amount content in the sample *C*_S_
Blank corrected sample signal intensity divided by the sensitivity (calibration slope) of the measurement procedure: $C_{S}=\frac{{\bar{J}}_{S}-{\bar{J}}_{B}}{F}$
2. Normalised signal intensity for the sample ${\bar{J}}_{S}$
a. Normalised signal intensity for the sample accounting for all sources of uncertainty: ${\bar{J}}_{S}={\bar{J}}_{R\_S}\cdot \delta_{rep\_S}\cdot \delta_{stab\_S}$
b. Average normalised raw signal intensity for consecutive replicates: ${\bar{J}}_{R\_S}=\frac{1}{n}\sum_{i}\frac{I_{S\_i}}{m_{S\_i}}$
3. Normalised signal intensity for the analytical blank ${\bar{J}}_{B}$
a. Normalised signal intensity for the analytical blank accounting for all sources of uncertainty: ${\bar{J}}_{B}={\bar{J}}_{R\_B}\cdot \delta_{\text{stab}\_B}\cdot \delta_{\text{rep}\_B}\cdot \delta_{\text{matrix}\_B}$
b. Average normalised raw signal intensity for consecutive replicates under closed sample line conditions: ${\bar{J}}_{R\_B}=\frac{1}{n}\sum_{i}\frac{I_{B\_i}}{{\bar{m}}_{S}}$
4. Calibration slope *F*
a. Slope accounting for all sources of uncertainty: $F=F_{\text{reg}}\cdot \delta_{\text{matrix}\_\text{std}}$
b. Slope of least squares regression line of the normalised signal intensity vs. the amount added Fe: $F_{\text{reg}}=\frac{r\sum C_{std\_j}\cdot {\bar{J}}_{std\_j}-\sum C_{\text{std}\_j}\cdot \sum {\bar{J}}_{\text{std}\_j}}{r\sum C_{std\;\_\;j}{}^{2}-{\left(\sum C_{\text{std}\;\_\;j}\right)}^{2}}$
5. Amount content of the added Fe in the calibration standards
a. Added Fe amount in the calibration standard: $C_{std\_j}=\frac{m_{\text{stock}\_j}}{\left(m_{\text{stock}\;\_\;j}+m_{\text{calSW}\;\_\;j}\right)}\cdot C_{\text{stock}}$
b. Amount in the stock solution: $C_{\text{stock}}=\frac{m_{\text{mother}\_\text{aliquot}}}{\left(m_{\text{stock}}\;+\;m_{\text{mother}\;\_\;\text{aliquot}}\right)}\cdot C_{\text{mother}}$

Parameter		Index	
*C*	Fe amount content (nmol kg^−1^)	S	Sample
*I*	Signal intensity (V)	B	Blank
$\bar{J}$	Average mass normalised intensity (V kg^−1^)	R	Raw
		Std	Calibration Standard
*F*	Sensitivity coefficient (slope, V nmol^−1^)	stock	Intermediate Fe standard stock solution (prepared dilution of the mother solution)
*n*	Number of replicates	mother	Mother Fe standard solution (commercial standard)
*r*	Number of calibration standards	i	Index referring to the *x* ^th^ sample replicate
*m* & $\bar{m}$	Mass and average mass (kg)	j	Index referring to the *x* ^th^ standard
		Reg	Sensitivity coefficient (calibration slope) obtained by linear regression
		calSW	a “low iron” seawater substrate used to produce the calibration curves
*δ*	Unity multiplicative correction factors carrying the relative uncertainty associated to the parameter considered	stab	Accounts for the uncertainty arising from the intensity stability over an analytical sequence
		matrix	Accounts for the uncertainty arising from matrix effects on the sensitivity
		rep	Accounts for the uncertainty arising from the intensity repeatability
		WtoV	Accounts for the uncertainty related to the difference in loaded mass whether it is done by weighing or volumetrically

#### Mass normalisation of the measurement signal

**Figure 2:**
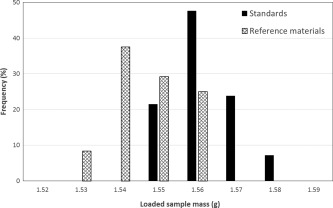
Frequency of variation (in %) of loaded masses for reference materials and calibration standards during the “reference material experiment.”

In most flow analysis methods incorporating a pre‐concentration column, the amount of sample loaded is assumed to remain the same for constant loading times and the resulting peak height signals (expressed in V) are used for the calculations. Variations in the loaded mass are thus not corrected for. However, this was found to be an issue as variations in sample mass were observed to be significant during the 32 h long “stability experiment,” with about 5% decrease in the sample mass loaded from the first to the last measurement (data not shown). During the “reference material experiment” the average loaded mass for samples (which were all run at the end of the sequence) was lower than for the standards (Fig. 2). The observed changes in mass loaded over time are likely to be due to changes in manifold parameters such as flow rates and column hydrodynamics. These results show the importance of weighing the amount of seawater loaded each time and of normalising the peak signal (symbol I, in V) to the loaded mass (in kg). In addition, gravimetric measurement, coupled with calibration of the analytical balance, provides tighter traceability to SI (the kg) of the amounts of loaded samples than loading by volumetric means.

As a result of this finding, mass normalised signals (symbol J, in V kg^−1^) were used throughout this study for the calculations (Eq. 2b; Table 2). Following the example given in Quétel et al. (2001), in Eqs. 2a, 3a, 4a, and 6 unity multiplicative factors were introduced to carry standard uncertainties associated with signal stability, matrix effects and differences during mass loadings. Applying the rules of uncertainty propagation to calculations involving several intensity results (such as average calculations, for instance), and thus combining together repeatability values associated to every single signal intensities, could lead to an overestimation of the resulting combined uncertainty. To avoid this risk, instead of applying the rules of uncertainty propagation to these calculations, these calculation results were multiplied by factors equated to 1 and carrying relative uncertainty estimations considered realistic and representative for these calculations. The ways these relative combined uncertainty components were estimated are discussed in the “Assessing the standard uncertainties” section below.

#### Blank corrections

Assessment of overall blank levels that reflect the reality of sample contamination during the measurement procedure is necessary. In the international inter‐laboratory comparison exercise IRONAGES, blanks were reported to range between 6% and 290% of the Fe content in the seawater sample (Petrov et al. 2007). Moreover, participants had diverse ways of defining and assessing their blanks (Bowie et al. 2006) and were, therefore, possibly overlooking different aspects of the contamination process. Sources of contamination during FI‐CL measurements include the Fe present in reagents (i.e., the added H_2_O_2,_ HCl, the buffer and rinse solutions and the chemiluminescence reagents) and Fe leaching from laboratory ware and parts of the experimental set‐up. Sample manipulations could also be a major contributor to the analytical blank as was shown to be the case by Petrov et al. (2007) during isotope dilution inductively coupled plasma mass spectrometry measurements using co‐precipitation with magnesium hydroxide for sample preparation. The Fe from the reagents used to generate the chemiluminescence reaction is included in the baseline. Baseline subtraction for the determination of net peak height or peak area signals, as commonly applied in FI methods, should therefore remove this possible bias. The influence of additions of chemical reagents for the purpose of preserving and/or conditioning the samples prior to the measurements (e.g., acid, H_2_O_2_) can be assessed using double spiking of the reagents. Previous studies using FI‐CL have shown their contribution to be low/negligible if care is taken to select high purity reagents (Bowie et al. 2003, 2004; Klunder et al. 2011). The major contribution to any blank signal arising from the reagents is therefore most likely to come from the buffer solution.

Descriptions of what a blank may represent are available from the International Union of Pure and Applied Chemistry (IUPAC). A ‘‘procedural blank’’ is ‘‘*where the analytical procedure is executed in all respects apart from the addition of the test portion’’* (McNaught and Wilkinson 1997; Inczedy et al. 1998). In this work using FI‐CL the procedural blank was considered to be the signal obtained with the “closed sample line” method as described above, i.e., loading only buffer (Bowie et al. 2004; Ussher et al. 2010a). Alternative measurement procedures for blank determination are the field blank approach (which requires a matrix containing no analyte) or varying sample loading times and extrapolating back to time zero (not accounting for the buffer solution) (Bowie et al. 2004). There is a risk that matrix effects and pH changes could influence final results due to fluctuations in the blank values determined using the “closed sample line” method and this is discussed further in the “Uncertainty on blank corrections” section below.

Normalised signal intensities were calculated by division by the average loaded sample mass (Eq. 3b). These blank values were 50–100 times lower than the signals for the seawater samples. Unity multiplicative correction factors were used to propagate uncertainties on stability and matrix effects (Eq. 3a) and are discussed in more detail in the “Assessing the standard uncertainties” section below.

#### Calculation of the calibration slope

**Figure 3:**
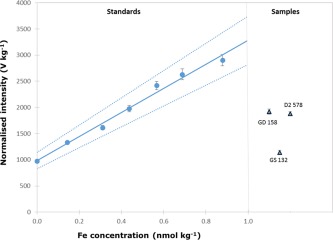
Unweighted calibration using average data for the regression. Blue dotted lines delimit a 95% confidence interval around the regression graph. Signal intensities observed for samples GD158, GS132 and D2578 are also reported.

The FI‐CL method has a different sensitivity for seawater than for ultra‐pure water because of matrix related effects (Bucciarelli et al. 2001). Thus, a common approach for the calibration under matrix‐matching conditions is to use a low level Fe seawater and fortify it with increasing amounts of Fe (Bucciarelli et al. 2001; Bowie et al. 2004; Ussher et al. 2010a; Klunder et al. 2011). In this work, in addition to the low level seawater alone (termed the “zero” standard), six calibration standards were prepared with Fe amount content ranging from 0.15 nmol kg^−1^ to 0.9 nmol kg^−1^. Since measurements were repeated six times for each calibration point, a total of 7 × 6 = 42 results were obtained. A linear regression was plotted (not shown), with the masses of Fe loaded (in kg, obtained by multiplication of the standard Fe mass fraction by the loaded mass of the replicate) on the *x* axis and the corresponding measured signal intensities (in V) on the *y* axis. The “behaviour” of the data was nearly the same irrespective of the scale of observation, with replicate results spread randomly around the regression graph in more or less the same way for all six standards prepared and tested. Common practice is to produce 3–4 replicates per Fe level and work with average values. Thus, a more practical way of establishing the calibration curve consists of plotting a linear regression between the group of 6 + 1 Fe amount content (C, in nmol kg^−1^) on the *x* axis and the corresponding average normalised intensities (J, in V kg^−1^) on the *y* axis (Fig. 3). The sensitivity coefficient (F, in V nmol^−1^), i.e., the slope, is obtained using Eq. 4b from Table 2. Weighted regression can also be performed but the calculations are more complex. In a weighted regression the higher the uncertainty on a *y* value the smaller the contribution of the *y* value to the regression slope. This is especially important if the increase of values on the *x* axis can be related to an increase of the standard uncertainty on corresponding values on the *y* axis. There was no difference with this dataset at the 95% confidence level between weighted and unweighted regressions. This is probably because the increase in the standard uncertainty with increased normalised intensity is limited. The comparison between these two approaches is further discussed in the next section.

### Assessing the standard uncertainties

Individual uncertainty components and the factors influencing their standard uncertainties were evaluated. This is necessary to enable a combined uncertainty estimation of the Fe amount content results.

#### Uncertainty on mass normalised measurement signals

**Figure 4:**
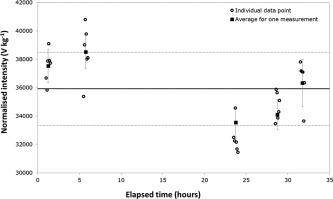
Stability over the 32 h “stability experiment’’ with seawater C using mass normalised peak height based results. Vertical bars indicate the standard deviation of the average of the six replicates. Horizontal lines indicate the average and standard deviations for the groups of five repeat measurements.

The repeatability (short term signal stability) of mass‐normalised intensities (peak height based signals) for one measurement varied between 1.9% and 4.0% RSD (relative standard deviation, *n* = 6) during the “reference material experiment” and between 2.4% and 4.9% RSD (*n* = 6) during the “stability experiment.” These variations in RSD cannot be explained by variations in the sample since variable RSD was also observed in the “stability experiment” where the same solution was measured. Together with a short term source of variability a longer term component, with the within‐sequence stability, was also involved and influencing the intensity values (Fig. 4). Over the 32 h long analytical sequence there was no clear trend, and as a result correction for drift was not possible. Therefore, the approach proposed is to estimate typical values for both components from the outcome of an ANOVA analysis and multiply the sample average mass normalised intensities by unity correction factors carrying the uncertainty for these two components (*δ*_rep_S_ and *δ*_stab_S_). Applying ANOVA to data from the “stability experiment” gave 4.1% and 6.3% as, respectively, the intensity repeatability and the relative within‐sequence‐stability component. Assuming independence between the intensity values used to calculate both types of RSDs, the relative standard uncertainties associated to *δ*_rep_S_ and *δ*_stab_S_ were estimated using these RSDs divided by square root 6 (the number of intensity replicates per measurement) and square root 5 (the number of repeat measurements in 32 h) respectively, to give values of 1.7% and 2.8%. Previously published work (Ussher et al. 2005) suggests that the major source of this uncertainty is associated with the column (loading and elution).

Sample loading and standard preparation cannot be performed gravimetrically on board ship and therefore this is done volumetrically, which may cause additional sources of uncertainty. In this case, the set of equations described in Table 2 will change slightly and result in Eq. (6) as described below:

(6) $$C_{S}=\frac{{\bar{I}}_{R\_S}\cdot \delta_{\text{rep}\_S}\cdot \delta_{\text{stab}\_S}\cdot \delta_{WtoV\_S}-{\bar{I}}_{R\_B}\cdot \delta_{\text{stab}\_B}\cdot \delta_{\text{rep}\_B}\cdot \delta_{\text{matrix}\_B}}{F_{\text{reg}}\cdot \delta_{\text{matrix}\_\text{std}}}$$

As a consequence of not using mass normalization, the sensitivity factor is determined by regression of the intensity (expressed in V) with the concentration (nmol L^−1^) and has the units V/nmol L^−1^. Secondly, an extra unity multiplicative correction factor (*δ*_WtoV_S_) was introduced to take account of the difference in the mass loading between samples and standards (Fig. 2). Using this dataset and assuming constant loading (i.e., without mass normalisation) its contribution to the final uncertainty budget was a few percent. Lastly, although the same approach can be used to quantify the uncertainty on the unity multiplicative factors corresponding to the intensity repeatability and within‐sequence‐stability component, the uncertainties will be higher than in the case of mass normalization. It must be noted that the within‐sequence‐stability during on‐board measurements might be different than in controlled laboratory conditions, but a specific assessment was not possible within the time frame of this study.

#### Uncertainty on blank corrections

The evaluation of the uncertainty on blank measurement signals was approached in a similar way as for the sample measurement signals. ANOVA analysis of the “stability experiment” results indicated 6.9% and 10% respectively for the intensity repeatability (*n* = 6) and the within‐sequence‐stability component (*n* = 5). A unity multiplicative factor *δ*_matrix_B_ with a value of 1.0 ± 0.2 was conservatively applied in Eq. 3a to account for the matrix differences between the blank samples and the standards used for calibration purposes. However, since the signal intensity for the procedural blank was about 50–100 lower than the intensity for the seawater samples in this project, this source of uncertainty on the blank correction had no influence on the combined uncertainties estimated for the Fe amount content in the samples investigated.

#### Uncertainty on the calibration slope

As discussed above, there are different statistical approaches that can be used to calculate the slope of the regression line (Miller 1991; Press 2012). Values obtained using different regression approaches are not significantly different at the 95% confident interval, but associated standard uncertainties do vary (Table 3). The standard uncertainty on the slope when using average normalised intensity values is the same whether the regression is weighted or unweighted. It is lower when using all individual data in the unweighted regression because there are more data points that follow a normal distribution. The importance of the number of standards and replicates on the size of the estimated standard uncertainty of the slope was studied. In Table 4 it can be seen that the number of standards used is a more important criterion than the number of replicates, but nevertheless the uncertainty on the sensitivity factor also improves using six rather than three replicates.

**Table 3:** Slopes and their associated standard uncertainties depending on the regression calculations considered. *r* is the number of standards and *n* the number of replicates per standard.

Regression approach		Data points	Sensitivity coefficient (=slope) (*F*)	
			Value	Uncertainty (*k* = 1)
Weighted regression		7 (*r*)	2301	83
Unweighted regression	Average values	7 (*r*)	2297	118
	All individual data	42 (r*n)	2297	56

**Table 4:** Dependence of the relative standard uncertainty (rsu) on the calculated slope/sensitivity coefficient, rsu (*F*), in %, on the number of replicates or calibration standards used.

n	rsu (F), with n = number of replicates using seven calibration standards (original + 6 Fe addition levels)	rsu (F), with n = number of calibration standards using six replicates for each standard
6	6.6	6.6
5	7.5	6.8
4	7.9	11.5
3	8.6	14.6

Small matrix differences between the three seawaters tested in the “matrix experiment” (*see* Table 1 for salinities) did not lead to significant differences (*t*_calc_ < 1.96 for all comparisons; *p* = 0.05) between the slopes obtained for SWA, SWB, and SWC. Iron binding ligands may also affect the linearity of calibration depending on the time allowed for equilibration after spiking seawater with Fe. The concentrations of iron binding ligands were not measured in these matrices and literature data give a range of ≈ 0.4–5.0 nM for Atlantic waters (Gledhill and Buck 2012). However, since SWC, D2, GS and GD were acidified to pH 1.7/1.8 the organic ligands should not impact on the calibration for these matrices (Lohan et al. 2006). Therefore, no uncertainty factor for differences in the calibrant matrix was applied.

## Discussion

### Application to seawater samples from the SAFe and GEOTRACES campaigns

Since consensus values are available for the Fe amount content in samples from the SAFe and GEOTRACES campaigns (GEOTRACES 2013), these data were compared with results obtained by application of the model for combined uncertainty estimation and the calculations described above. Samples D2, GS, and GD were analyzed using six replicates each time, the “closed sample line” approach for blank assessment and a least square regression calibration line with seven levels (no Fe added + 6 levels of added Fe) in SWA. This was the “reference material experiment,” and results obtained are reported in Table 5. Estimated expanded (coverage factor *k* = 2) relative combined uncertainties were around 12% on a peak height basis, and around 10% on a peak area basis. Using this dataset, the combined uncertainty was slightly higher using volumetric loading compared with gravimetric loading. For example, for sample GD the combined expanded uncertainty increased from 12% to 13% for peak height integration. It can be seen that both peak height and peak area based results are systematically lower than the consensus values. Results obtained for GS and GD (peak height and peak area basis) and peak area results for D2 were in agreement with consensus values within uncertainty statements. These conclusions were reached from the observation that the expanded combined uncertainty (*k* = 2) on the difference between a measured and the corresponding consensus value was greater than the difference itself in all cases (calculations according to a methodology reported in Linsinger 2010). For the peak height results for the D2 sample, the expanded uncertainty on the difference was smaller than the difference itself but only by less than 3%. These results validate the measurement procedure implemented and the uncertainty estimation process developed. They nevertheless point to the presence of a systematic effect not yet (sufficiently) corrected for.

**Table 5:** Amount content results with combined expanded uncertainty with a coverage factor (*k*) of 2 (i.e., 95% confidence interval) for the three sea water samples from the SAFe and GEOTRACES campaigns using gravimetric loading. Consensus values were downloaded from the GEOTRACES.org website and are from May 2013.

Sample		Obtained Fe amount content				Consensus Fe amount content	
	Peak height			Peak area			
	Value (nmol kg^−1^)		Relative uncertainty (%)	Value (nmol kg^−1^)	Relative uncertainty (%)	Value (nmol kg^−1^)	Relative uncertainty (%)
D2	0.82 ± 0.10		12	0.861 ± 0.086	10	0.933 ± 0.046	4.9
GS	0.478 ± 0.060		12	0.500 ± 0.051	10	0.546 ± 0.092	16.8
GD	0.800 ± 0.099		12	0.836 ± 0.084	10	1.0 ± 0.2	20.0

An overview of the values of the input parameters and their associated standard uncertainties for these experiments is given in Supporting Information Table S1. The relative contributions of the different input parameters to the uncertainty budget are given for sample GD in Table 6 as an example. The normalised signal intensity repeatability accounts for only 7.9% of the total uncertainty. The within‐sequence‐stability component (assessed over 32 h) and the uncertainty on the sensitivity coefficient (calibration slope) are the most important contributors to the combined uncertainty with relative contributions of 21.6% and 69.7%. Therefore, it is beneficial to have a low uncertainty on the calibration slope. For this reason, it is favourable to use sufficient replicates (6) and number of standards (at least the non‐spiked standards and five spiked levels, Table 4). Moreover, correctly estimating the within‐sequence‐stability is key and should be done under the same measurement conditions as for the samples.

**Table 6:** Relative contributions (%) to the combined uncertainty budget estimated for the dissolved Fe level measured by FI‐CL in the GD sample from the GEOTRACES campaign (symbols as in Table 2). The intermediate result refers to the parameters used in Eq. 1 of Table 2, in which all associated uncertainties are included. The GUM Workbench dedicated software package (Metrodata GmbH 2003) was used for the uncertainty propagation calculations.

Quantity		Gravimetric loading	
		Peak height	Peak area
Average normalised signal intensity for sample ${\bar{J}}_{S}$ (V kg^−1^)	Intermediate result	29.5	44.4
	${\bar{J}}_{R\_S}$ (treated as constant)	‐	‐
	$\delta_{\text{rep}\_S}$	7.9	9.4
	$\delta_{\text{stab}\_S}$	21.6	35.0
Average normalised signal intensity for blank ${\bar{J}}_{B}$ (V kg^−1^)	Intermediate result	0.6	1.4
	$I_{B}$ (treated as constant)	‐	‐
	${\bar{m}}_{S}$	0.0	0.0
	$\delta_{rep\_B}$	0.0	0.6
	$\delta_{\text{stab}\_B}$	0.1	0.0
	$\delta_{\text{matrix}\_B}$	0.5	0.8
Sensitivity coefficient (or slope) *F* (V nmol^−1^)	Intermediate result	69.7	54.3
	$F_{\text{reg}}$	69.7	54.3
	$\delta_{\text{matrix}\_\text{std}}$	0.0	0.0

Results obtained indicate that an uncertainty estimation based on the signal repeatability alone, as is often done in FI‐CL studies, is not a realistic estimation of the overall uncertainty of the procedure. However, taking into account only the major contributions, the combined expanded uncertainty could be approximated using Eq. (7):

(7) $$U(C_{s})\approx 2\cdot C_{S}\sqrt{\frac{{\bar{J}}_{S}{}^{2}\cdot \left[{\left(\frac{u(\delta_{\text{rep}\_S})}{\delta_{\text{rep}\_S}}\right)}^{2}+{\left(\frac{u(\delta_{stab\_S})}{\delta_{stab\_S}}\right)}^{2}\right]}{{\left({\bar{J}}_{S}-{\bar{J}}_{B}\right)}^{2}}+{\left(\frac{u(F)}{F}\right)}^{2}}$$

In this, the standard uncertainty on the intensity repeatability and within‐sequence‐stability can be assessed using ANOVA analyses of repeat measurements of the same solution. The uncertainty on the calibration slope can be obtained using statistical tools. This simplified approach assumes that the blank does not significantly contribute to the uncertainty and should therefore have a much lower intensity compared with the sample (as was the case in this study). When using data from this project the uncertainty obtained with Eq. (7) was nearly identical to the uncertainty calculated above (for example the difference was less than 0.2% for GD using peak height data). Therefore, if the assumptions are valid this simplified approach provides a realistic uncertainty estimate.

### Peak area vs. peak height

The bias between results and consensus values was around −12% for D2 and GS and −20% for GD, on a peak height basis, and around −8% for D2 and GS and −16% for GD, on a peak area basis. This also means that peak height results were systematically lower than the peak area results by approximately 4–5%. The cause is unlikely to be related to an error in the placement of the baseline for integration, as this affects height less than area (Dyson 1998). In contrast, the asymmetry of the FI‐CL peaks could be a possible source of error during peak height measurement, since peak area is less sensitive to peak asymmetry than peak height (Dyson 1998).

It can also be observed in Table 5 that estimated combined uncertainties are larger for peak height than for peak area based results. This is mainly related to a larger uncertainty associated with the sensitivity coefficient for peak height compared with peak area (Supporting Information Table S1). Area integration is considered the “true” measure of the amount of solute (Dyson 1998) and possible problems specific to peak area data such as peak overlap and/or low signal‐to‐noise ratios (Dyson 1998) are not an issue with FI‐CL measurements. These observations lead to the conclusion that peak area data may be preferable to peak height data with FI‐CL measurement results (at least for the FI‐CL manifold and chemistry described here), contrary to common practice. Additionally, users should routinely and systematically describe the way peak data are processed.

## Comments and recommendations

The amount content of dissolved Fe in marine waters is measured to elucidate the biogeochemical cycling of this element and its role in the oceanic sequestration of atmospheric CO_2_. However, quantifying the amount of Fe present in < 0.2 *μ*m filtered and acidified seawater samples remains a difficult analytical task, and achieving reliable results is a challenging objective. Moreover, the uncertainty as part of the measurement results is easily underestimated.

FI‐CL is a technique commonly applied because of its portability and hence suitability for shipboard deployment. From a technological perspective the use of piston pumps (such as micro‐sequential injection) has the potential to alleviate some of the issues highlighted in the present manuscript that are associated with the use of peristaltic pumps (Oliveira et al. 2015).

This paper proposes that the relative expanded (*k* = 2) combined uncertainty of the measurement results using FI‐CL in the described configuration cannot be better than about 10–15% for seawater samples containing 0.5–1 nmol kg^−1^ of dissolved Fe. When applied on‐board ship the minimum achievable uncertainty is likely to be even larger owing to the more challenging working conditions compared with shore‐based laboratories. Moreover, this paper emphasises the fact that it will be beneficial to researchers to refine measurement practices in order to improve the likelihood of achieving lower uncertainty targets. For FI‐CL, the uncertainties associated with the calibration slope and the within‐sequence‐stability are shown to be much greater sources of uncertainty than the intensity repeatability alone. Experimental planning must therefore systematically address the identification of strategies aimed at quantifying and minimising the role of these uncertainty contributors. These strategies include the use of as many calibration standards as possible (ideally five plus the “zero” standard measured with six replicates) and measurements repeated regularly for the same sample over the entire analytical sequence. In view of the long term instability observed during the “stability experiment” a practical recommendation is to analyse a check standard every 2 h and recalibrate if the value is outside of a specified range, e.g., ± 5%. It is also shown that more attention needs to be paid to the way FI‐CL peak data are collected and processed, as this could lead to significant errors with respect to the size of the combined uncertainties. To enhance the transparency of these aspects it is recommended that more comprehensive descriptions of the methods used to validate the measurement procedures (including the way peak data collection/processing is performed) are included in publications and reports. Moreover, a simple equation to approximately estimate the uncertainty has been proposed, which is valid if the blank levels are significantly lower than the levels of interest.

## Supporting information

Supporting Information
