# Supplementary material for: Combined uncertainty estimation for the determination of the dissolved iron amount content in seawater using flow injection with chemiluminescence detection
Source: Limnol Oceanogr Methods. 2015 Sep 18;13(12):673–86. doi: 10.1002/lom3.10057 (PMC4995610; doi:10.1002/lom3.10057)
Supplement: Supplementary file 1 — Supporting Information [file LOM3-13-673-s001.docx]

**Supplementary information**

Table S1: details of the uncertainty budget associated to the result of the measurement by FI-CL (with gravimetric loading) of the dissolved Fe amount content in the D2 reference material from SAFe. Symbols as in Table 2. The GUM Workbench dedicated software package (Metrodata GmbH, 2003) was used for the uncertainty propagation calculations.

| Quantity | | | Peak height | | | Peak area | | |
| --- | --- | --- | --- | --- | --- | --- | --- | --- |
|  |  |  | Value | Standard Uncertainty (k=1) | | Value | Standard Uncertainty (k=1) | |
|  |  |  |  | Absolute | % |  | Absolute | % |
| Average normalised signal intensity for sample  __  (V kg^-1^) | **Intermediate**  **result** | D2 | **1918** | **63** | **3.3** | **52179** | **1700** | **3.3** |
|  |  | GS | **1140** | **37** | **3.3** | **30809** | **1000** | **3.3** |
|  |  | GD | **1879** | **62** | **3.3** | **50711** | **1700** | **3.3** |
|  | **__** | D2 | 1918 | 0 | 0 | 52179 | 0 | 0 |
|  |  | GS | 1140 | 0 | 0 | 30809 | 0 | 0 |
|  |  | GD | 1879 | 0 | 0 | 50711 | 0 | 0 |
|  | **__** | | 1 | 0.017 | 1.7 | 1 | 0.015 | 1.5 |
|  | **__** | | 1 | 0.028 | 2.8 | 1 | 0.029 | 2.9 |
| Average normalised signal intensity for blank  __  (V kg^-1^) | **Intermediate result** | | **41.8** | **2.2** | **5.3** | **1115** | **190** | **17.0** |
|  | __ | | 0.0645 | 0 | 0 | 1.72 | 0 | 0.0 |
|  | __ | | 0.001542 | 0.000004 | 0.3 | 0.001542 | 0.000004 | 0.3 |
|  | __ | | 1 | 0.069 | 6.9 | 1 | 0.17 | 17.0 |
|  | __ | | 1 | 0.10 | 10 | 1 | 0 | 0 |
|  | __ | | 1 | 0.2 | 20 | 1 | 0.2 | 20 |
| Sensitivity coefficient (or slope)  F  (V nmol^-1^) | **Intermediate result** | | **2297** | **118** | **5.1** | **59330** | **2190** | **3.7** |
|  | $F_{reg}$ | | 2297 | 118 | 5.1 | 59330 | 2190 | 3.7 |
|  | $\delta_{matrix\_std}$ | | 1 | 0 | 0 | 1 | 0 | 0 |
